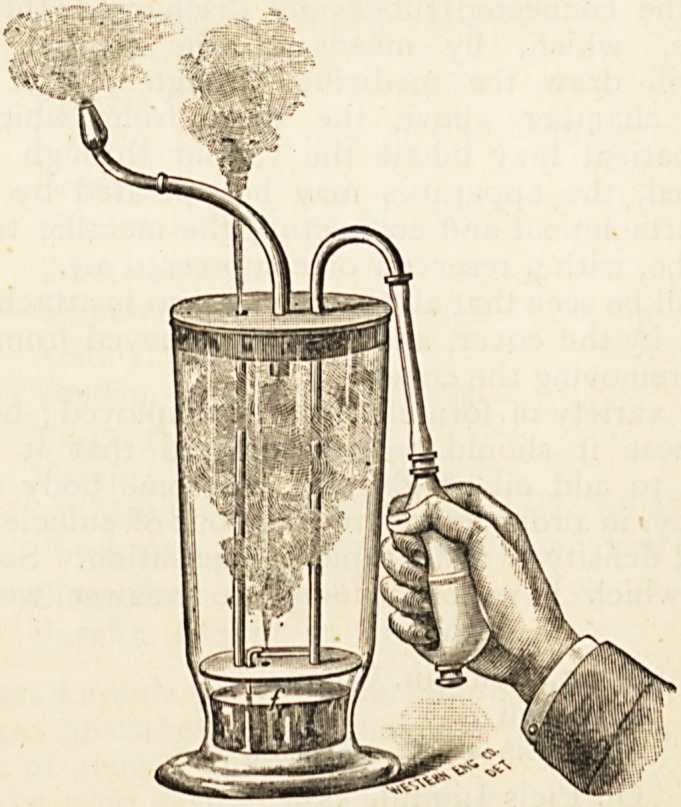# Notes on Preparations for the Sick

**Published:** 1887-09

**Authors:** 


### Notes on Preparations for the Sick.

### Report on some Meat-Extracts.

We have recently enquired into the dietetic and medicinal value of the following preparations :—

**Valentine's Meat Juice.**
**Murdoch's Liquid Food.**
**Bush's Bovinine.**

The first of these, **Valentine's Juice,** is now a well-known and much valued restorative in conditions of great exhaustion. It is a fluid of a blood-red colour, having ·04 per cent. of the amount of colour in blood : it is of syrupy consistence, readily soluble in water, of neutral reaction, and perfectly free from sediment. Its spectrum gives two lines, one on the *E* side of *D*, the other on the *F* side of *E*, answering to the characteristics of hæmochromogen (Stokes's reduced hæmatin). It contains coagulable albumen in solution amounting to about 7.0 per cent. ; nitrogenous extractives soluble in alcohol, 11.6 per cent. ; matters insoluble in alcohol, including inorganic salts, about 9 per cent. ; and water 72.4.

Summary :—

| | |
|---|---|
| Water . . . . . . . . | 72.4 |
| Coagulable Albumen . . . . | 7. |
| Salts and Extractives . . . . | 9. |
| Extractives Soluble in Alcohol . . | 11.6 |
| | 100.0 |

The aqueous solution has an agreeable taste, little digestion is necessary, and its composition shows that its value for blood-making must be very high. A fluid with seven per cent. of soluble albumen is something more than a mere stimulating restorative ; it supplies available nutriment for blood-making in an easily assimilated form. Few patients dislike it, and these dislike all other similar foods for the sick : we have found it to be of the highest dietetic value, and it may be well spoken of as the most perfect of beef-preparations.

**Murdoch's Liquid Food** is a blood-red fluid, containing much granular sediment, of crystals and disintegrated blood corpuscles; its reaction is neutral, and its specific gravity is 1030. Its spectrum shows one thin distinct line on the *C* side of *D*, and a broad fainter line on the *E* side of *D*, corresponding nearly to that of hæmoglobin, except that the *D* line is too near the red end of the spectrum. The amount of colouring matter is .20 that of blood. It contains 15.6 per cent. of coagulable albumen, and only traces of nitrogenous extractives and salts.

Summary:—

| | |
|---|---|
| Water . . . . . . . . | 83.4 |
| Coagulable Albumen . . . . | 15.6 |
| Extractives and Salts insoluble in Alcohol | .4 |
| Matters Soluble in Alcohol . . . . | .6 |
| | 100.0 |

Analysis and physical examination show it to be a solution of hæmoglobin with much insoluble granular sediment. In comparison with the Valentine juice it has a larger proportion of water and more than twice the proportion of soluble albumen, but with a great deficiency of extractives and salts.

This large proportion of albumen should give it a very high nutritive value, but it has less of the stimulating restorative effect in proportion to the relatively smaller amount of nitrogenous metabolites and salines. Such a solution of hæmoglobin with soluble albumen ought theoretically to be the best of blood-making nutriment, and experience shows it to be so, when the patient is able to take large quantities of the fluid: the colour, smell, taste, and after-effects of the fluid are however so nauseating and repulsive to most patients that few can be persuaded to persevere with it long. We have tried it in various forms: it is most readily taken in beef-tea, but the medicine-bottle in combination with liquorice and chloroform-water has been the most satisfactory method.

In the treatment of anæmia we have sometimes been successful: in one case the hæmoglobin rose from 45 to 60 per cent. in the course of fifteen days with first one drachm and then two drachms of Murdoch's Food every four hours: the convalescence was however much more rapid after sulphate of iron was given. In another case the hæmoglobin continued steadily at 30 per cent. for a whole month in spite of two-drachm doses every four hours, whereas within two weeks after the commencement of sulphate of iron the hæmoglobin had risen to 65 per cent.

It has been said that four tablespoonfuls daily will make eight per cent. of new blood weekly: our experience has not been quite in accord with this; it certainly is inferior to iron in hæmatinic value, but nevertheless it has potent dietetic and medicinal properties which ought to be, and are often found to be, of the greatest value. One patient found that she could not get on without it, and frequently had to resume her "Murdoch" after discontinuing it. It has been in the malnutrition of infants we have been most successful with this food. Even where peptonised milk and other prepared foods seemed to be failing, the addition of five or more drops to the milk at each administration has several times been followed by almost immediate improvement and steady recovery.

There is certainly scope for improvement in the preparation of this so-called "Food." True, it may be a typical food for the rectum, and we have frequently given it peptonised with milk by enemata; even then it should not be prepared in the room, as the odour is so disgusting. But the mere sight of the thick muddy-looking blood-red fluid is sufficient to raise a prejudice against it, which the removal of the cork will only intensify.

**Bush's Bovinine** is a dark-coloured liquid containing little sediment; the amount of colouring matter, estimated by the globinometer, being .20 that of healthy blood. Its spectrum gives a well-marked band at *C*, with a fainter band at *D*, resembling that given by an alkaline solution of hæmatin. The fluid is neutral in reaction, and contains 18.2 per cent. of coagulable albumen. The nitrogenous extractives insoluble in alcohol, and including the inorganic salts (phosphates, sulphates, and chlorides of potassium, sodium, and calcium), amount to .6 per cent. The nitrogenous extractives soluble in excess of alcohol amount to .45 per cent.

Summary:—

| | |
|---|---|
| Water . . . . . . . | 80.75 |
| Albumen . . . . . . . | 18.2 |
| Inorganic Salts . . . . . | .6 |
| Nitrogenous Extractives . . . | .45 |
| | 100.00 |

Its analysis shows it to have a high potential value as a nutriment for the sick: less stimulating than the "Valentine," more nutritious than the "Murdoch," whilst its appearance and odour are superior to the latter, and patients take it more readily. A fluid containing one-fifth of the colouring matter of blood, with eighteen per cent. of soluble albumen, and one per cent. of extractives and salines, ought to be excellent material from which to manufacture new blood. The digestion of such a fluid appears to take away from its apparent value, inasmuch as the hæmoglobin is decomposed and the hæmatin passes through the intestine in an insoluble condition; nevertheless, we are inclined to think that this fluid is likely to become better known and more extensively employed than at present: its dietetic value is undoubtedly great.

---

**Pharmaceutical Preparations.** BURGOYNE, BURBIDGES, CYRIAX AND FARRIES, London.

**Moxon's Effervescent Magnesian Aperient** is said to be decidedly the most agreeable purgative ever offered to the public. Possibly a mere sight of the label and the paper in which the bottle is wrapped might have the desired effect: should this fail, the effervescing powder forms in water an agreeable drink, which by its antacid, febrifuge, and aperient qualities must frequently be of service in a great variety of functional disorders where a saline aperient is indicated.

**Dr. Koch's Meat-Peptone.**—This preparation appears to be one of the most satisfactory of the numerous attempts hitherto made to peptonise the albuminous constituents of ordinary food. The product is put up in jellied form, also in tablets and in lozenges. It is quite soluble, dissolving readily on the tongue; has an agreeable taste, and great nutritive value. It must be easily absorbed and assimilated, whether given by mouth or rectum; and as it requires no digestion, must be of value where the digestion is impaired, or as a rapid restorative for travellers and others who cannot obtain regular nourishment. A box of the meat-peptone-lozenges, containing 25 to 30 grammes, will contain sufficient for a day's journey without other nourishment.

**Hazen Morse's Maltopepsyn.**—This preparation is a combination of pepsine, pancreatine, acid lactophosphate of lime, and dried extract of malt. It therefore combines the action of the digestive juices of the salivary glands, stomach, and pancreas; and is said to be capable of digesting all aliment taken by mankind.

The saccharated pepsine is that from the pig, freed from mucus and all impurities; and the malt extract is made from Canada barley, carefully selected, and which has the deserved reputation of being the best barley in the world.

The combination is agreeable to the palate, odourless, active, stable, and inexpensive.

**Peptoleine.**—This is the name given to one of the many emulsions of cod liver oil, which are provided for those who find a difficulty in taking or digesting the oil in the unmixed form. It is one of the most palatable of these combinations, has little of the unpleasant taste of the oil, leaves little after-taste, is readily miscible with water or milk, is easily digested, is said to be of equal nutritive value to the undiluted cod liver oil, and contains fifty per cent. of the latter.

We have also received from this firm a **Semple's Atomizing Inhaler,** which is described as the most perfect apparatus that has ever been presented for the treatment of diseases of the respiratory tract by medicated vapour.

It consists of an atomizer, working inside a chamber, which forms a reservoir, in which the fine spray is retained, and from which it may be drawn into the respiratory tract at will.

"From a study of the above cut, it will be seen that Semple's Atomizing Inhaler consists of a glass container covered with a metallic plate, to which is attached a cork stopper. Into this is inserted a metallic tube for inhalation, with a bulb attachment for insertion into the mouth or the nostrils. A pendant disk is supported from the cover by two tubes; one, the air tube, passes through the cover and disk and is open from end to end. The other is closed at the bottom and passes through the cover, the portion exterior to the container being bent as indicated, and having attached to it a rubber tube with syringe bulb. Branching from the tube is a small tube closed at its outer end and having a small orifice on top, and just in front of and at right angles to a similar orifice in the end of the bent tube *f*. These two tubes are soldered together for mutual support, and the latter passes loosely through one of the holes in the disk, and extends to near the bottom of the container. This tube is attached to the disk on one side of the hole through which it passes to strengthen the parts.

"For using the inhaler, place enough of the desired inhalant in the bottom of the jar to cover the lower end of the tube *f*, taking care that the pendant disk is not submerged. By compressing the bulb air is forced through the connected tubes and discharged through the jet orifice, which, by means of the relation of parts shown, will draw the medicine through *f* and vaporize it in the chamber above the disk, from which chamber the patient may inhale the vapour through the tube. If preferred, the apparatus may be operated by removing the bulb attachment and connecting the metallic tubes by a rubber tube, with a reservoir of compressed air.

"It will be seen that all the mechanism is attached to and supported by the cover, and may be removed from the container by removing the cover."

A great variety of formulæ may be employed; but in preparing these it should be remembered that it is always necessary to add oil or glycerine or some body of similar consistency, in order to secure a vapour of sufficient permanence and density to resist rapid condensation. Some of the formulæ which have been found to answer well are as follows:

℞. Tinct. Benzoin. Comp.
Glycerini
Alcoholis āā ℥i—Misce.

℞. Ol. Picis Liquid. ʒss.

Vaselin. Liquid. ℥j—Misce.

℞. Ol. Eucalypti ʒj

Vaselin. Liquid. ℥j—Misce.

℞. Tinct. Iodi ʒiss

Glycerini ℥j

Alcoholis q. s. ad ℥iij—Misce.

℞. Tinct. Iodi ʒij

Acid. Carbolic. ʒij

Ext. Tolutani fl. ℥j

Glycerini ℥j

Alcoholis q. s. ad ℥iij—Misce.

℞. Ext. Cubebæ fl.

Ext. Tolutani sol. fl.

Tinct. Iodi

Tinct. Camphoræ

Acid. Carbolic. āā ʒj

Glycerini ℥i⅓

Alcoholis q. s. ad ℥iv.—Misce.

℞. Ext. Stramonii sem. fl.

Ext. Hyoscyami fl. āā ʒj

Ext. Belladonnæ fl. ʒss

Glycerini ℥j

Alcoholis q. s. ad ℥iij—Misce.

℞. Bals. Copaibæ ℥ss

Etheris ℥j

Vaselin. Liquid. q. s. ad ℥iv—Misce.

℞. Etheris

Vaselin. Liquid. āā ℥j—Misce.

℞. Tinct. Iodi ʒss

Glycerini ℥ss—Misce.

℞. Acid. Carbolic. gr. x.

Vaselin. Liquid. ℥j—Misce.

℞. Tinct. Benzoin. Comp. ℥j
Vaselin. Liquid. ℥ij—Misce.
Mix the two fluids: evaporate off the alcohol by gentle heat, filter—yields two ounces.

℞. Chloroform.

Vaselin. Liquid. āā ℥j—Misce.

This last formula provides an admirable method of producing local anæsthesia, and may also be utilised for the production of general anæsthesia.

We have used several of the above formulæ, and have found them to answer admirably: we quite endorse the statement that this apparatus is the best ever presented for treatment by medicated vapour.

There can be no question that by the use of this inhaler a fine spray of various active drugs can be introduced far down the respiratory tract. By a series of deep inspirations the lungs become so charged with the medicated vapour in so fine a spray that much of it is not deposited on the mucous membrane, but may be seen to escape on exhalation. These medicinal substances do clearly find their way far down: much of the spray remains, whilst a portion is exhaled again. We have thus a means by which medicinal bodies are brought into contact with a large area of respiratory mucous membrane on which they may exert a local influence, and through which they may reach the blood and the tissues of the body in general but of the lungs in particular. If inhalation is the thing, surely here we have the best of inhalers: whatever good is to be obtained by medication through the respiratory tract can best be obtained by such a machine as this. The apparatus is so devised that in the process of converting the medicine into vapour all the coarser globules are thrown against the sides of the container and condensed, while the most finely subdivided particles remain suspended in the atmosphere as a dense vapour until drawn into the air-passages, where the greater portion is arrested and retained, whilst a smaller portion is again discharged. The stability of the vapour is such that it will remain for several minutes, more or less, according to the density of the inhalant used, before condensing, and thus admit of thorough respiration and secure intimate contact of the medicament with the mucous membrane of the finest air-passages.

The range of application of this method of medication is a wide one, and must often be preferable to medication through the gastric mucous membrane: the respiratory membrane appears to be equally, if not better, adapted for the easy and rapid absorption of medicines than is that of the stomach or rectum: accordingly the method may be of use in the treatment of general diseases; it is, however, for the local treatment of nasal and pulmonary catarrh, laryngitis, membranous croup, hay-fever, diphtheria, bronchitis, pneumonia, asthma, and phthisis that the apparatus becomes of special, if not indispensable, value.

In the last-mentioned disease it may be utilised for the introduction of such drugs as iodoform, iodol, terebene, creasote, which, in solution and combination with liquid vaseline, appear to give rise to little irritation.

**Pancreatized Cod Liver Oil.** B. Keen, Bristol.

This preparation is said to be nearly tasteless. It does not produce the discomfort and nausea so often occasioned by the oil itself.

---

**Marshalls' Preparations of Wheat.** James & Thomas Marshall, Glasgow.

These articles appear to be superior in nutritive value to the usual farinaceous foods. The starch-foods, apart from the starch they contain, derive their nutritive value from the milk in which they are cooked. These wheat-preparations can be employed for all the purposes to which starchy foods have been applied, but require no adventitious aid, inasmuch as they are themselves of great nutritive value.

**Farola** is a highly refined form of wheat-flour, in which no cellulose or irritating impurities remain. It is described as "the fat of kidneys of wheat," and as the "extract or essence of wheat." It is light, easy of digestion, pleasing to the palate, vieing with the finest arrowroot in elegance of appearance, whilst it is far superior in nutritive value.

**Tritola** is a food resembling Semolina in appearance, but differing chiefly in the larger size of its granules. The contents of the wheat-berry, after removal of the epidermis, are broken down into pearl-like granules, which preserve all the strength, flavour, and nutriment of the grain when cooked. Tritola naturally moulds itself into a firm and yet friable shape, which readily breaks down under the action of the digestive solvents.

**Granola** consists of the whole pure contents of the grain in a free granular state, with the exception of the germ. It contains a certain amount of disintegrated bran, and is intended to meet the wants of those who require an intestinal stimulant, more particularly those of sedentary habits. It contains all the elements necessary to the constitution of a perfect food.

The **Semolina** of this firm is the best part of the contents of the wheat-grain with all the husk removed, and in a free granular state: it is the product of a very high class wheat, is highly nutritious, palatable, and easily digested. It contains all the constituents of the best bread, and its granular shape fits it for a great variety of purposes in cookery for which flour or bread is not so well adapted.

Their **Oat Flour** is made from selected oats grown on the richest lands of the Lothians. The coarse and irritating parts of the grain are removed; what is retained is the pure flour, containing all that is of value for food.

All these preparations are capable of being cooked in a variety of ways for the table: recipes are given with each packet.

---

**Coca Wine.** Armbrecht, Nelson, & Co., London.

The Erythroxylon Coca, growing on the mountains of Bolivia and Peru, is now so well known as a powerful nerve stimulant as to need no description. It is now generally admitted that Coca strengthens the mental and physical powers, allays fatigue, abolishes hunger, and assuages thirst. In small doses it increases appetite and promotes digestion; it relieves the headache of nervous debility, and may be given with benefit in cases of opium and morphia habit. Armbrecht's preparation is made with Burgundy or Malaga, and one wineglassful represents one drachm of the leaves. We have found it of service in the early morning exhaustion of old people, and in removing the feeling of fatigue and exhaustion during the hot weather of the present summer.

## Figures and Tables

**Figure f1:**